# P-69. What if? Opportunities for IV to PO Antibiotic Transition in Bone and Joint Infection Treatment

**DOI:** 10.1093/ofid/ofae631.276

**Published:** 2025-01-29

**Authors:** Graham Price, Makeisha Dance, Patrick E H Jackson, Corey Medler

**Affiliations:** Virginia Commonwealth University School of Pharmacy, Charlotesville, Virginia; Virginia Commonwealth University School of Pharmacy, Charlotesville, Virginia; University of Virginia School of Medicine, Charlotesville, Virginia; University of Virginia Health , Charlottesville, Virginia

## Abstract

**Background:**

Historically, bone and joint infections have been treated with intravenous (IV) antimicrobials. IV to oral (PO) antibiotic transition implementation differs among institutions and prescribers. This practice has increased in recent years as new literature has demonstrated comparable efficacy of PO and IV treatment. Use of outpatient IV therapy often necessitates using a central line. This introduces additional risks, particularly line-related complications such as clots and central line infections. The purpose of this project was to identify opportunities for transitioning from IV to PO therapy in patients discharged on IV antibiotics for bone/joint infections at an academic medical center.

Baseline Patient Characteristics

**Figure:**
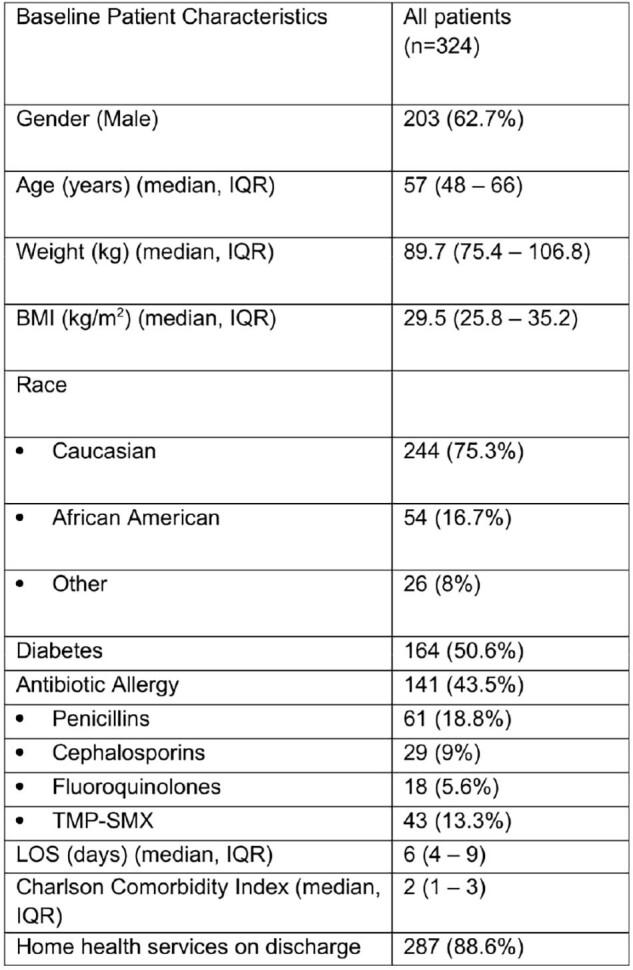

**Methods:**

We performed a retrospective, cross-sectional study of adults discharged on IV antibiotics between January 2020 and December 2023. Inclusion criteria included age ≥ 18 years, patient followed by institutional outpatient parenteral antimicrobial therapy service, and receipt of at least one IV antibiotic for a bone/joint infection. Patients with axial osteomyelitis were excluded. The primary outcome was the percentage of patients with the potential to transition from IV to PO antibiotics. Secondary outcomes included incidence of adverse drug events (ADE).

**Results:**

324 patients were included. Osteomyelitis was the most common infection (48.5%) followed by joint infection (27.5%). Source control procedures were performed for 83% of patients. Cultures were positive in 75% of patients, and *Staphylococcus* species were the most common pathogens (45%). In total, 237 (73.1%) of patients qualified for a complete PO regimen. Half of patients (50.3%) received at least one PO antibiotic as part of their discharge therapy. Antibiotic changes occurred after discharge in 15.4% of patients, and 21% of patients experienced an antibiotic associated ADE.

**Conclusion:**

Most patients discharged on IV antibiotics for bone/joint infection at this medical center could have received a complete PO regimen. While PO antibiotics are not benign, there is the potential to reduce adverse effects. These findings highlight opportunities for IV to PO transition at discharge and the potential to reduce line associated complications.

**Disclosures:**

**Patrick E H Jackson, MD**, Gentiva Hospice: spouse employment|Kindred Hospice: spouse employment|Pfizer: Grant/Research Support

